# Neuro-Oncology Patients as Human Research Subjects: Ethical Considerations for Cognitive and Behavioral Testing for Research Purposes

**DOI:** 10.3390/cancers14030692

**Published:** 2022-01-29

**Authors:** Jasleen Kaur, Andrew Egladyous, Claudia Valdivia, Andy G. S. Daniel, Saritha Krishna, Alexander A. Aabedi, David Brang, Shawn L. Hervey-Jumper

**Affiliations:** 1 Department of Neurological Surgery, University of California, San Francisco, CA 94143, USA; jasleen.kaur4@ucsf.edu (J.K.); aye7@rwjms.rutgers.edu (A.E.); ccvaldiv@iu.edu (C.V.); andy.daniel@ucsf.edu (A.G.S.D.); Saritha.Krishna@ucsf.edu (S.K.); alexander.aabedi@ucsf.edu (A.A.A.); 2 Department of Psychology, University of Michigan, Ann Arbor, MI 48109, USA; djbrang@umich.edu; 3 Helen Diller Family Comprehensive Cancer Center, 1450, 3rd Street, San Francisco, CA 94158, USA

**Keywords:** glioma, cognition, speech, behavior, language, aphasia, neuro-oncology, ethical, anxiety, investigational

## Abstract

**Simple Summary:**

Previous publications have elaborated on the exposure of ethical issues surrounding the enrollment and neurological testing of brain cancer patients into clinical studies. Existing literature has been tailored to provide insight on how to overcome ethical challenges for clinical team members but not for the research component that runs in parallel. The aim of this paper is to highlight the obstacles that researchers encounter when obtaining informed consent and administering language, cognitive or behavioral tasks for the sole purpose of research. Researchers should be encouraged to practice their best judgment and effectively communicate the purpose of the study while emphasizing the voluntary participation of neurologically impaired cancer patients. The solutions proposed in this paper can serve as future reference and a guide on maintaining a transparent balance between research and clinical testing for both researchers and clinical team members in the neuro-oncology field.

**Abstract:**

Language, cognition, and behavioral testing have become a fundamental component of standard clinical care for brain cancer patients. Many existing publications have identified and addressed potential ethical issues that are present in the biomedical setting mostly centering around the enrollment of vulnerable populations for therapeutic clinical trials. Well-established guides and publications have served as useful tools for clinicians; however, little has been published for researchers who share the same stage but administer tests and collect valuable data solely for non-therapeutic investigational purposes derived from voluntary patient participation. Obtaining informed consent and administering language, cognition, and behavioral tasks for the sole purpose of research involving cancer patients that exhibit motor speech difficulties and cognitive impairments has its own hardships. Researchers may encounter patients who experience emotional responses during tasks that challenge their existing impairments. Patients may have difficulty differentiating between clinical testing and research testing due to similarity of task design and their physician’s dual role as a principal investigator in the study. It is important for researchers to practice the proposed methods emphasized in this article to maintain the overall well-being of patients while simultaneously fulfilling the purpose of the study in a research setting.

## 1. Introduction and Overview

Brain cancers exist within the context of non-neoplastic neurons and glia. Neoplastic cell proliferation may therefore directly impact neurological and cognitive processes [1]. The neurological and cognitive implications of central nervous system (CNS) tumors have become an important area of investigation [2]. The primary goal being to better understand how impairments caused by intrinsic and extrinsic brain tumors differ from those caused by lesions impacting the CNS structures (i.e., multiple sclerosis, spinal cord injury, and dementia). A clearer understanding of tumor molecular classification has resulted in the enhanced ability to predict long(er) term survivors. Therefore, disease-free survival periods may be marked by lasting neurological impairments due to either the tumor itself or sequelae of oncological treatments. For this reason, detailed patient testing of functional domains, together with health-related quality of life patient reported outcomes have shed light on both disease status as well as functional status for patients. Longitudinal cognitive and behavioral testing is underway at many institutions to understand symptom and disease trajectory. Cognition testing, however, may be obtained (1) as part of standard of care patient management, (2) for investigational only purpose, or (3) collected for clinical purposes yet maintained in a registry for investigational use. Challenges such as managing patient anxieties, deciding on the optimal time to administer a behavioral task, task duration, and consenting aphasic and severely cognitively impaired patients each require special consideration [3,4].

In 1979, the newly formed National Commission for the Protection of Human Subjects of Biomedical and Behavioral Research gathered in response to concerns over unethical studies and published the Belmont Report [5]. This publication, along with published recommendations from institutional review boards (IRBs) across the United States, continue to serve as an indispensable guide for how research, including both biomedical and behavioral research, should be conducted while maximally protecting the rights of participants in parallel. Today the abundance of ethical guides, in the form of articles, manuals, checklists, definitions, videos, and mandatory human subject’s research trainings are a source of references for researchers to help guide the design and implementation of ethical research. However, much of what has been published concentrates on ethical considerations for the traditional components of biomedical research (i.e., clinical trials, genetic testing, and tissue sampling). Much less has been written about the ethical considerations involving language, cognitive and behavioral testing for purely investigational purposes for patients with cancer.

Though the IRB publishes a vast amount of content on research ethics, the principal role of the IRB is to ensure that research under its jurisdiction adheres to the highest standards of ethical principles and legal regulations. Studies that may involve vulnerable patient populations include scenarios in which cognitive and behavioral data are obtained for both clinical use as well as investigational purposes; however, the specific use and application of this data may be poorly defined for the patient [4]. More specifically, cognition testing may be obtained for clinical use with data recorded prospectively for investigation. Alternatively, cognition testing which is not necessary or intended for clinical use may be confusing for both patients and families. Similarly, cognitive, and behavioral impairments can trigger emotional responses in patients during testing, which may lead to challenges in completing tasks and obtaining accurate data. Methods to minimize anxiety and frustration patients face during language, cognitive, and behavioral testing may need to be employed. Attempting to protect the physical and emotional welfare of patients is by no means a simple task. The clinical concern must be balanced against scientific rigor given that, if the study team were to exclude patients with even mild cognitive impairments this would skew study results and limit the generalizability of scientific findings.

The decision to participate in biomedical research is made at a very vulnerable time for the patient—often within several days of being diagnosed with a significant medical condition (e.g., brain tumor) [6]. Neurocognitive tests are often integrated in pediatric protocols for central nervous system tumors; however, this commentary intends to discuss examples of ethical challenges and solutions encountered during cognitive research studies in the adult neuro-oncology context. Our goal is to highlight challenges, potential solutions, and best practices, to ensure that patients with brain cancer who participate in biomedical cognition research receive optimal protection while studies are conducted with the best possible scientific rigor.

## 2. Informed Consent

**Figure 1:**
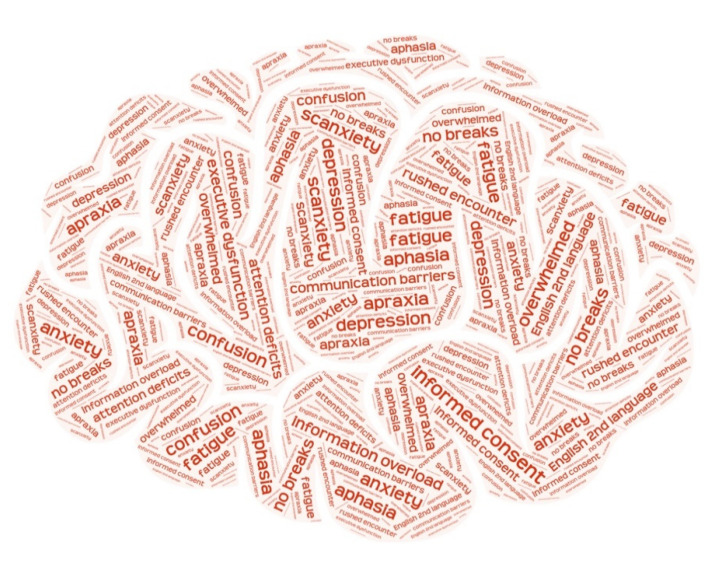
Word cloud of competing factors for neuro-oncology patients participating in cognition research.

A fundamental requirement of all research with human subjects is the assurance that informed consent has been obtained from all study participants. Informed consent, defined as the willingness to participate based on a complete understanding of the risks, benefits, and purpose of the research, is sometimes difficult to obtain [7]. Cognitive research for patients with glioma is subject to challenges stemming from two broad categories: (1) the neuro-oncology context of the study and the (2) communicative and cognitive impairments demonstrated by the patients. These two sources each provide their separate ethical dilemmas that merit broader discussion (Figure 1).

### 2.1. Neuro-Oncology Context

In general, cognitive dysfunction is defined as an impairment in one or more cognitive domains [8]. An impairment in at least one cognitive domain is seen in the majority of patients with intrinsic brain tumors such as low and high-grade gliomas [9]. Cognitive impairments experienced by cancer patients are associated with lower rates of return to work and poor quality of life therefore investigations focused on speech, cognition, and behavior have direct implications on clinical outcomes [8,9,10,11,12]. Patients who are considering enrollment in a non-therapeutic clinical research study, in which their doctor has a dual role as a treating physician and principal investigator, require special considerations. Often, patients are first introduced to the research study by their treating clinician (i.e., neurosurgeon or neuro-oncologist) during the same appointment at which treatment details are discussed. From the perspective of the patient, the volume of information received during a clinical visit can be a barrier to fully understanding informational study materials. Adding research consents during clinical visits combined with details about surgical procedures, chemoradiation, and symptom management discussions may result in information overload. Furthermore, the subject matter itself may be uncomfortable for patients given the personal and professional stigma associated with uncovering cognitive limitations.

After a patient expresses interest in a voluntary research study, an appointment is scheduled with a clinical research coordinator to discuss the logistics of enrollment, sign consent documents, and undergo cognitive, language and behavioral testing. At times these encounters can be rushed given competing scheduled events such as imaging studies, lab draws, and discussions about next steps in clinical care. Cognitive and behavioral research testing may happen a few days before surgical intervention or before starting cancer directed therapies to ensure that data is collected close to defined clinical benchmarks. By the time a patient has their research testing appointment, they may feel exhausted by any mandatory clinical related testing, particularly if research testing duplicates or is similar in nature to standard of care clinical testing. Back-to-back clinical care appointments without breaks for medications, a meal, and travelling from one appointment location to another may contribute to fatigue and lack of interest in participation for voluntary research.

Details of the research study and study consent may be addressed by either a clinical research coordinator or a clinician involved in the study. Clinical team members who are in the immediate care group may be the best resource to introduce the study given their expertise in the field, clinical background, and the familiarity with the patient’s medical records and history [8]. However, in the setting in which the patient’s clinician is the principal investigator of the study, there may be unintentional pressure on the patient to participate to be compliant with their doctor. Furthermore, it is possible that patients may believe that they will be more closely monitored or offered the best possible treatment available as result of their study participation. To combat this possible influence, it is essential that patients clearly understand that their clinical care will remain unchanged regardless of their study participation.

### 2.2. Language and Cognitive Impairments

In the adult neuro-oncology patient population, over 70% of patients experience cognitive, language, or behavioral impairments, which negatively impacts outcomes [8,9,10,11,12]. Therefore, obtaining informed research consent may pose a challenge. While severe cognitive dysfunction prohibits study enrollment, even subtle changes to one’s ability to understand spoken or written language requires special consideration when obtaining informed consent for clinical or research purposes [3,4,13]. If completed in haste, discussion of study details with patients with aphasia may leave them with inadequate time to express their understanding of the research. Though they may be able to understand the description of the study, they may have difficulty inquiring about the technical research aspects of the study. This difficulty could cause patients to avoid asking questions in fear of embarrassment or increased anxiety. Thus, when working with patients with cognitive and language impairments, it is essential to employ teach-back consenting methods that ensure a complete understanding of the study [14]. Distinguishing between comprehension and recall may also be a challenge for researchers during the informed consent process and cognitive testing. All patients should demonstrate a thorough understanding of the research before consenting to participation. 

In addition to aphasia (loss of speech), some patients may experience speech impairments like apraxia (impaired speech production) or dysarthria (slurred speech), which can impair motor planning and muscle strength necessary to articulate and produce sounds. These impairments, similar to expressive aphasias, can also discourage patients from posing questions [13]. The onset of many speech and language impairments is often a recent and novel experience, given the tumor’s violation of dorsal and ventral language pathways. Therefore, patients may not have developed coping strategies to compensate for these communication barriers.

## 3. Drawing the Line between Clinical Care and Research

In most settings, cognitive and language testing of neuro-oncology patients is administered as part of, or to supplement, the standard of clinical care. It can, therefore, be complicated for some patients to distinguish between appointments, interactions, and procedures that relate to clinical care versus those that relate to research. When patients meet for testing with clinical research coordinators, they may also engage with radiologists, nurses, physician associates, oncologists, neurophysiologists, neurologists, and surgeons. Many times, neuro-oncology cognitive research can compete with therapeutic clinical research. Therefore, research tests are most often administered at dedicated appointments, separate from those required for clinical care. The increased amount of patient interaction with health care professionals also increases the amount of information a patient receives regarding their medical care. Therefore, in some instances information overload may occur resulting in patients asking clinical questions to researchers who are unable to adequately answer the questions, as they are outside of their area of expertise. A high frequency of interaction can blur the lines of what tasks are part of clinical care versus research tasks that are not required and therefore, may not directly benefit patients [3,15].

Furthermore, appointments are often conducted in the same clinic rooms used for standard of care clinical encounters. Though this is beneficial for reducing travel across a large medical campus and legitimizing research aims, it also may hinder a patient’s ability to discern between standard of care and research [16,17]. Additional to the time and space overlap, there is a similarity in the content of research testing tasks. The overlapping content of activities allows for a more seamless experience for the patient in which the same events provide information for both the individual patient and the research study.

In addition to the challenges of distinguishing between clinical care and research, researchers may question the validity of results that are confounded by emotional and behavioral factors. One such example is the experimental confounds posed by research subject anxiety. There is an abundance of evidence demonstrating the impact of patient anxiety on cognitive task performance. Experiencing anxiety can influence task performance depending on the difficulty of the cognitive testing, therefore, increased anxiety will decrease task performance on harder tasks [18,19,20]. The validity of results can be impacted by patients who are unable to complete tasks due to not understanding task instructions, physical limitations that interfere with using devices that tasks may require and task designs that trigger emotional and behavioral responses. Therefore, it is important for researchers to differentiate if task performance is impacted by tumor-related neurophysiological dysfunction or if it is anxiety related.

## 4. Emotional Distress

To better understand how language and cognition are impacted by infiltrative intrinsic and extrinsic brain tumors, patients are asked to complete tasks that assess their functional capacity across several essential domains. However, testing cancer patients in an impaired task domain can cause emotional distress. Given the context of a recently diagnosed brain tumor or recent onset of tumor-related symptoms, testing patients in areas of impaired function comes with a possibility of exacerbating anxiety, frustration, and other adverse emotions. These complex emotions are likely to be experienced regardless of whether the patient completes the research task or not, but it is possible that asking patients to perform these tasks catalyzes the emotional response. Patients having trouble articulating words or sentences during language testing can feel perplexed as they may be able to visualize and comprehend the stimuli shown but are unable to exercise their motor speech. Techniques to provide support and encouragement to complete testing should be executed by the research team member administering these tasks to avoid any adverse events caused by triggering emotional distress. Patients experiencing cognitive impairments may feel rushed when doing tasks that are administered at normal speeds which can produce a sense of incapability and anxiety. Therefore, the emotional context of cognition and behavioral research must be considered and managed when working with brain tumor patients.

## 5. Proposals

In order to ensure compassionate and ethical cognitive and language studies in the adult neuro-oncology patient population, we propose solutions that supplement current practices. Following informed consent, the teach-back method is a useful technique [5]. When obtaining consent in patients with aphasia, it may be challenging to utilize the teach-back method [4,12]. One may consider augmenting the teach-back process with the addition of a checklist that includes salient topics that the patient needs to exemplify understanding. Essential components of the checklist could include: (1) verification that the patient understands that refusing to participate in the research does not change their clinical care; (2) the patient being knowledgeable about specific aspects of cognition testing which are for research purposes versus those which are for clinical care; (3) the patient knowing at what points temporally during their care and treatment that the research components begin and end; (4) the patient understanding that the research will most likely have no direct benefit to them (Table 1) [15]. The goal would be to have each consideration checked off, and any items that have not been discussed on the checklist be readdressed to ensure the patient and their proxy understand. A different way to approach the teach-back method for patients with aphasia who maintain intact motor function would be to have them write down any important key points that illustrate the purpose of the study and what their rights and roles are as a study participant. Patients should be reminded that their participation is completely voluntary, and they may opt out at any time if participation becomes overwhelming. Furthermore, study personnel may consider revisiting a patient’s decision to remain enrolled in research especially during periods of emotional instability or disease progression.

To make it transparent for patients to distinguish between clinical and research appointments, the patient’s appointment itinerary should clearly state the title of their appointment as research appointment, brief description of the voluntary study, and point of contact if they chose to withdraw their interest due to exhaustion that may result from previous clinical appointments or time restrictions. If a patient has multiple clinical appointments in a one-day period and they are interested in participating in research, it could be beneficial to break down the research testing into multiple parts depending on study design and at what time-point cognitive and behavioral tasks need to be administered. For example, cognitive tasks and language testing can be administered as a priority after which other elements such as questionnaires and surveys can be administered at a more convenient time.

Neuro-oncology patients with tumors within the dominant hemisphere perisylvian language network may have a difficult time reading the consent forms. It may, therefore, be useful to add an executive summary statement to the end of the consent form highlighting important takeaways the patients should understand. Visual aids in the form of a video or picture may also be created and shown to the patients to supplement the consenting process. A complementary option may include the presence of a health care proxy to witness the consenting process and ask questions to facilitate communication on the behalf of the research participant. It is important to also have the proxy sign the witness section of the informed consent form and both the proxy and research participant be provided a copy of all documents signed for their records. If a patient is experiencing weakness or numbness in their dominant hand which limits their ability to sign and date the informed consent, a proxy or advocate can sign as witness on their behalf. If the patient can fully understand informed consent but has this limitation, the researcher can verbally record their consent with permission.

To ensure successful communication and comprehension between patient and the person obtaining informed consent, it may be helpful to utilize techniques that can simplify but not take away the purpose of the study such as repetition of key concepts, speaking slowly with the appropriate tone, pausing between key points, asking simplified yes or no questions and writing key words [21]. Researchers can distinguish between a patient’s ability to comprehend and recalling by listening to see if patient is repeating the information verbatim or if the patient is able to express their understanding of the research study and their role as a participant in different terms.

An increasing number of clinical trial protocols now include cognitive and health related quality of life primary and secondary endpoints. As a result, patients may be enrolled in both clinical trials and nontherapeutic clinical cognition research [22]. In this setting, clinical trials with therapeutic potential should take priority over nontherapeutic clinical research studies. This may involve discussions between study teams and even halting nontherapeutic clinical research protocols to avoid testing fatigue, missed appointments, and patient confusion. Due to time sensitive situations or any factors that may compromise a patient’s health, if the principal investigator is also the treating clinician, using their best judgement, they have the choice to change the patient’s participation status to “principal investigator withdrawal” for the study. Before a researcher approaches a patient that may be experiencing cognitive and language impairments, it can be helpful to ask a clinical care team member if the patient should be approached for enrollment. It is often in the best interest of the patient to be first assessed by a clinician prior to allowing researchers to proceed with the study for only cognition testing.

In the intraoperative setting, it may be of value to preface any cognition task with a statement that recording of passively recorded clinical data is entirely optional. Additionally, any passively acquired data for investigational purposes must not prolong standard of care interventions. In situations in which investigation only tasks are applied as part of the study, researchers should consider determining a specific time slot for such testing and participants must be reminded that this portion of the study is optional. A time cap for any language, cognition, or behavioral task timing should be considered and tailored in a way which will include all testing needs as well as regulate the time a patient is awake for reasons outside of clinical necessity [23].

**Figure 2:**
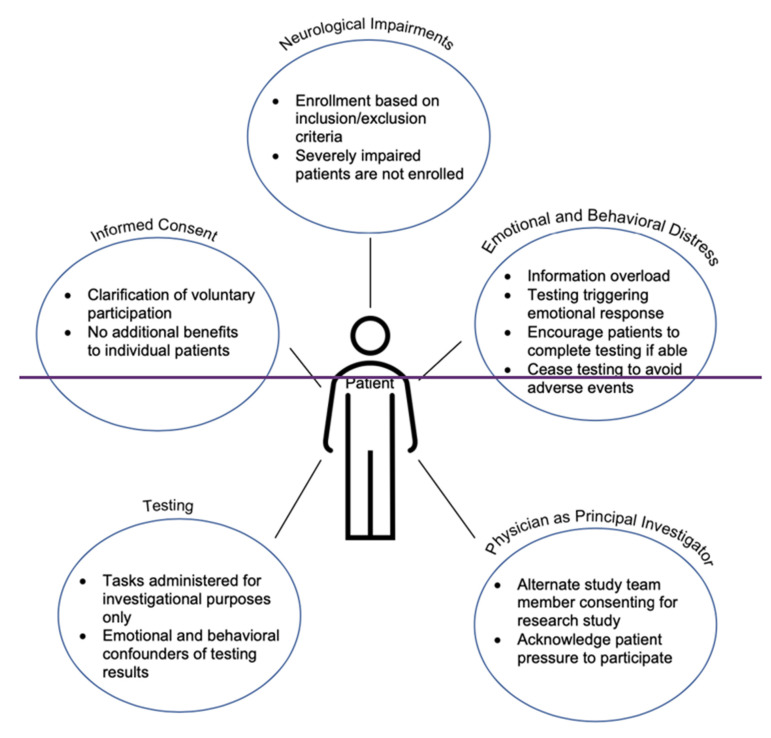
Summary of non-clinical research testing in the neuro-oncology setting.

One of the best practices when approaching research-only cognition and behavioral testing for patients with cancer is to remind them that their participation is voluntary. Before starting any cognitive tasks or language tasks, it should be clear to the patient whether they are participating in standard of care treatment or investigational testing. Patients should know that that breaks, pauses, or discontinuation of the study are all acceptable options. It is important to remind patients to do their best but to not be discouraged with their performance because uncovering impairments is exactly what testing is designed to capture (Figure 2).

Researchers must remain vigilant in their awareness that a research study is secondary to clinical concerns for vulnerable populations, even when necessary compromises introduce limitations to a study (e.g., greater attrition in non-clinical populations, potential confounds added by the testing environment, and patient fatigue). Accordingly, researchers must faithfully report notable protocol deviations if they introduce confounds or alternative interpretations of the data and researchers should design studies to minimize the impact of these issues. For example, limiting the total duration of testing sessions, allowing breaks between tasks, and reducing the speed of the task can reduce patient fatigue. To minimize emotional distress, researchers and/or the tasks can provide positive feedback throughout the session; relatedly, for the same reason, tasks that give the patient the appearance that they are failing at a high should be avoided. Finally, task designs can minimize the impact of potential confounds, such as using a mixed-design instead of a blocked-design when possible, to circumvent condition differences due to fatigue or emotional distress.

## 6. Conclusions

Investigations focused on the interactions between brain tumors and functional circuits in the human brain requires detailed interrogation of the CNS in the form of cognition testing. Patients with cancer, however, have important emotional, neurological, and cognitive considerations (Table 2). Although often difficult, best practices exist to ensure balance between the rigorous study of this important topic with compassionate care for patient.

## Figures and Tables

**Table 1:** Teach-Back Checklist for Cognition and Behavioral Testing.

Cognition Testing Checklist
◊ Verification by patient that participation is not mandatory
◊ Clear distinction that each visit is for research, clinical care, or both
◊ Clear distinction within encounter for exactly when investigational testing begins
◊ Confirmation to patient of no direct benefit for participation
◊ Confirmation to patient of no penalty for not participating

**Table 2:** Summary of ethical challenges and solutions for neuro-oncology researchers.

Ethical Challenges	Solutions
Unintentional pressure from physician if the physician is the principal investigator of the research	Clarify when completing informed consent that care and treatment will not be impacted if patient decides not to participate in voluntary research
Getting patients to complete cognitive and language testing when experiencing emotional distress and testing fatigue	Make it clear that it is okay to stop testing at any time Design cognitive tasks that can assess the same domains at different speeds Prime the patient regarding task length and task instructions
Obtaining informed consent from patients exhibiting aphasia and other cognitive deficits	Implement the teach-back method Checklist that distinguishes between research and clinical aspect of their care Have health proxy sign as witness Provide visual aids to supplement consenting process Approach patient after clearance with their physician Communicate by repeating key concepts, speaking slowly, and asking yes or no questions
Time constraints during intraoperative setting for research testing	Assign specific time slot for testing Shorten tasks to minimize the time a patent is awake
